# Folding scene investigation: membrane proteins

**DOI:** 10.1016/j.sbi.2008.12.005

**Published:** 2009-02

**Authors:** Paula J Booth, Paul Curnow

**Affiliations:** Department of Biochemistry, University of Bristol, University Walk, Bristol BS8 1TD, UK

## Abstract

Investigations into protein folding have concentrated on experimentally tractable proteins with the result that membrane protein folding remains unsolved. New evidence is providing insight into the nature of the interactions stabilising the folded state of α-helical membrane proteins as well as giving hints on the character of the folding transition state. These developments show that classical methods used for water-soluble proteins can be successfully adapted for membrane proteins. The advances, coupled with increasing numbers of solved crystal structures, augur well for future research into the mechanisms of membrane protein folding.

## Introduction

Investigations into the folding of integral membrane proteins have been severely hampered by a number of factors [1,2]. Proteins that reside in biological membranes have very different surface properties to water-soluble proteins. Membrane proteins expose hydrophobic surfaces to the membrane interior, whilst polar and charged amino acids lie on the protein exterior that interacts with membrane lipid headgroups and the aqueous regions at either side of a membrane. The proteins are also susceptible to the lateral forces and elastic properties of their surrounding lipid bilayer [3]. Mimicking these complex solvent interactions to maintain the folded, functional state of a membrane protein is a major barrier in molecular structural and functional studies [1]. Furthermore, many membrane proteins are large and consist of more than one domain or subunit. They also frequently possess dynamic structures because of the conformational flexibility required to transduce signals or transport substances across a membrane.

New approaches are required to solve the membrane protein folding problem and some of these, which are based on manipulating the lipid bilayer, are beginning to prove very fruitful [4]. Additionally, evidence is emerging that skilful adaptations of classical folding methods, developed on small water-soluble proteins, are also very effective. In order to understand a mechanism fully, a combination of kinetic and thermodynamic investigations is required. Fortunately, this approach is now proving feasible for helical membrane proteins. In a previous review [5^••^] we highlighted the exciting possibilities for such study and here we see them coming to fruition. This current review focuses on enlightening recent investigations into the folding mechanisms of integral membrane proteins with α-helical structures.

## Reversible folding and linear free energy relationships

A particularly successful method to measure the free energy of folding is through reversible chemical denaturation. The equilibrium constant for folding is readily obtained for a microscopically reversible two-state system, making it straightforward to derive the free energy change associated with the reaction. The relationship between free energy and denaturant concentration is generally linear for water-soluble proteins, which enables extrapolation to determine the free energy of folding in the absence of denaturant.

Membrane protein work is plagued by irreversible denaturation and protein aggregation. However, there have been hints for some time that reversible folding is possible and that some folding reactions can be fitted by a two-state transition with a linear dependence of free energy on denaturant [1,6–11]. It has now been definitively shown that a folding reaction of an α-helical membrane protein (bacteriorhodopsin, bR) follows a microscopically reversible, two-state process [12]. bR reversibly unfolds upon the addition of SDS to mixed lipid, detergent micelles (of 1,2-dimyristoyl-*sn*-glycero-3-phosphocholine, DMPC and 3-[(3-cholamidopropyl)dimethylammonio]-1-propanesulfonate, CHAPS). Overall, this is a very complex folding reaction involving a number of intermediate states [13–15]. Conditions have been established for a cooperative, reversible two-state reaction between a partly unfolded SDS state and folded bR; a reaction that represents the major, final folding step of the protein [12]. Linear free energy relationships were observed in both equilibrium and kinetic data. The logarithms of the measured unfolding and folding rate constants are each linear with SDS mole fraction, and combining this rate data generates a classical chevron plot. This analysis is very familiar in water-soluble protein folding studies, but this is the first case of such a plot for a membrane protein.

Many interesting pieces of information result from these linear relationships. An unexpectedly slow unfolding rate in the absence of denaturant is revealed for bR [12]. This is illuminating since membrane proteins are frequently assumed to be unstable outside their native membrane, yet this suggests that bR has a very high kinetic stability *in vitro*. Indeed the protein would not unfold during the course of its lifetime, which if true *in vivo*, would preclude any damaging misfolding occurring in the membrane. Moreover, there are cases where mutant membrane proteins reach the cell membrane, but are linked with late onset of disease [16]. A relatively non-disruptive mutation could give a folded protein in the membrane, able to function for some time before gradually unfolding or degrading; thus delaying the onset of malfunction and disease.

## Comparisons with water-soluble proteins

There are three membrane proteins where folding has been characterised to the point where a comparison of folding free energy can be made. These are bR, *Escherichia coli* diacylglycerol kinase (DGK) and the *Streptococcus lividans* potassium channel (KcsA) [9,12,17]. Here, we compare these membrane proteins to water-soluble proteins that fold by two-state and three-state kinetics. For the latter we use a previously published representative folding dataset [18] expanded to include some additional information on larger monomers and oligomers. We do not distinguish between the denaturant used (either urea or guanidinium hydrochloride) or oligomeric state.

**Figure 1:**
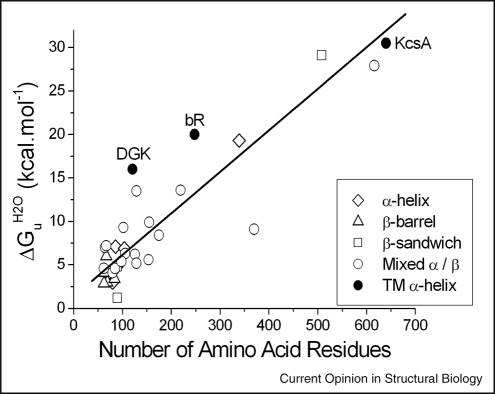
Comparison of the free energies of unfolding, per amino acid residue, for integral membrane proteins and water-soluble proteins. $\Delta G_{\text{u}}^{{\text{H}}_{2}\text{O}}$ values for the integral membrane proteins bR, DGK and KcsA are broadly consistent with values obtained for water-soluble proteins. Data for water-soluble proteins are taken mainly from [18] and grouped into different structural categories. All three membrane proteins are dominated by helical structure. bR is a monomer, DGK a trimer (although the free energy here is for a monomer unfolding transition) and KcsA a tetramer. Thus in this latter case the number of amino acids relates to this oligomeric state. The solid line is an arbitrary guide for the eye.

Intriguingly, it appears that the overall free energy change of unfolding in the absence of denaturant ($\Delta G_{\text{u}}^{{\text{H}}_{2}\text{O}}$) for water-soluble and membrane proteins scales similarly with protein size (Figure 1). The hydrophobic nature of membrane proteins and low dielectric environment within the membrane imply that the forces which stabilise membrane proteins differ to those for water-soluble proteins. In fact, it seems that both types of protein are equally stable on a per residue basis and thus it is the balance of weak forces, rather than the nature of those forces *per se*, that is important in determining stability. Further information on the nature of the stabilising interactions comes from a recent study on bR. The dogma surrounding membrane protein folding suggests that hydrogen bonds will be important stabilising interactions and that these will be stronger than hydrogen bonds in water-soluble proteins, because of the low dielectric of the membrane interior and absence of water molecules. Double mutant cycle analysis, using the SDS unfolding assay to determine free energies, has shown this is not the case [19^••^]. Hydrogen bonds between bR helices are quite weak, being about 0.6 kcal mol^−1^ and of similar length to those within water-soluble proteins.

The denaturants and solvent systems used in the three membrane protein studies discussed here are different to those usually employed for water-soluble proteins. The anionic detergent SDS was used to denature bR and DGK from mixed DMPC/CHAPS micelles or decylmaltoside (DM) micelles, respectively, whilst trifluoroethanol (TFE) was used to unfold KcsA from dodecylmaltoside (DDM) micelles. SDS and TFE will initially partition into the micelles, giving mixed DMPC/CHAPS/SDS, DM/SDS or DDM/TFE micelles, and then at higher concentrations form SDS micelles or TFE solutions that solubilise the denatured protein. It is interesting to investigate the response of the folding process to these denaturants, in comparison to that of urea in aqueous solutions. Clues can come from *m-*values which illustrate the magnitude of the linear dependence of the overall unfolding free energy on denaturant (i.e. from $\Delta {\text{G}}_{\text{u}}=\Delta {\text{G}}_{\text{u}}^{{\text{H}}_{2}\text{O}}+m_{\text{U}-\text{F}}\chi \text{SDS}$). For water-soluble proteins, overall *m*-values (*m*_U–F_) typically fall within the range 0.5–5 kcal mol^−1^ m^−1^. Unfolding KcsA with TFE gives *m*_U*–*F_ of 2.5 kcal mol^−1^ m^−1^, which is consistent with *m*-values for soluble proteins. However, it is less straightforward to make a comparison when using SDS in mixed micelles because bulk mole fraction is used as the measure of SDS concentration (giving *m*-values in units of kcal mol^−1^). It is also not currently possible to separate direct protein–SDS interactions from the effect of the altered properties of the DMPC/CHAPS/SDS micelle on the protein as SDS concentration is increased. For the membrane proteins unfolded in mixed micelles with SDS, *m*-values tend to be large, with *m*_U*–*F_ for monomeric bR and DGK being 25 and 22 kcal mol^−1^, respectively, or ∼0.1 and 0.18 kcal mol^−1^ per residue.

The chevron plot, showing the dependence of the folding and unfolding rate constants on denaturant, for bR is very asymmetric (see Figure 6, Ref. [12]) compared to most water-soluble proteins. The very slow unfolding rate of bR in the absence of denaturant reflects the steep dependency of this rate on SDS. The *m*_TS*–*F_ value relating to the gradient of this unfolding arm of the chevron plot line is thus large. The other arm of the chevron plot has a much shallower gradient (i.e. the folding *m*_TS*–*U_ value) showing there is a smaller dependence of the folding rate on SDS. Information on the folding transition state for membrane proteins is scarce, but this recent work on bR starts to give some insight [12]. Kinetic *m*-values can be used to give a measure of the position of the transition state with respect to the unfolded and folded states, through a *β*-value (calculated from the ratio *m*_TS–U_/*m*_TS–U_ + *m*_TS–F_ [20]). A low *β*-value of ∼0.1 was found for bR [12], which indicates that the transition state is closer to the unfolded than the folded state. For most water-soluble proteins *β* is higher, in the range 0.4–0.9, suggesting transition states closer to the folded state. However, in contrast to the relatively unstructured unfolded states of many water-soluble proteins, the unfolded state for the bR reaction is structured (with an α-helical content equivalent to ∼4, of the native 7, transmembrane α-helices; ∼130 of the 248 amino acids are in α-helical structures), and thus the transition state will also have a considerable degree of structure. This reinforces earlier suggestions that a critical helical core aids successful refolding of membrane proteins and that key interactions form early in folding from the SDS state [5^••^,21]. In light of extensive studies on bR which show that complete secondary structure formation precedes retinal binding [6,14,15,21,22], the transition state most likely involves formation of helical structure, probably accompanied by solvent reorganisation.

## Mechanical strength and unfolding under an applied force

Dynamic force microscopy can be used to measure the mechanical response of a particular region of a protein under an applied force. These are non-equilibrium measurements where the unfolding force depends on an activation barrier for that particular protein structural segment under the directional applied force. This situation is different to chemical unfolding [23,24] and mechanical strength is not related to the overall thermodynamic stability of a protein. bR has been the testing ground for mechanical unfolding experiments on membrane proteins [25–27]. Recently, further detail has emerged from forced unfolding studies of bR mutants [28]. Reductions were found in the activation barriers to the forced unfolding, which correlate with a decrease in the distance from the free energy minimum of the folded state to the transition state barrier. This is an example of Hammond behaviour; as the energy difference between two consecutive states in a reaction (such as the folded and transition states) is reduced, the two states become more similar in structure and closer on the reaction coordinate.

Forced-induced unfolding has also taken membrane protein work to another level by probing the roughness of the energy landscape. Proteins fold over multi-dimensional free energy surfaces, about which very little is known for proteins within lipid bilayer membranes. Forced unfolding enables the roughness of the energy surface to be probed [29]. The energy surfaces for individual helical unfolding in bR are found to be relatively rugged, being ∼5*k*_*B*_*T* [30^••^] and similar to that obtained for globular water-soluble proteins. This is an informative result in view of the very different solvent environments of the two types of proteins.

## Influence of the surrounding membrane

Membrane proteins are susceptible to changes in the properties of their surrounding native membrane lipids *in vivo*, or detergent and lipids *in vitro*. Whilst successes in membrane protein research are due to both judicious and serendipitous choices of detergents and lipids *in vitro*, guiding principles are beginning to emerge.

A variety of roles are increasingly being identified for both specific and non-specific interactions between proteins and lipids in folding, stability and function [1,3,31,32]. The incorporation of lipids in detergent micelles frequently increases protein stability and folding. Further evidence for this comes from the report that mixtures of phosphatidylglycerol (PG) and PE lipids added to DDM micelles seem to improve the recovery of KcsA tetramers from a denatured TFE state [17]. Moreover, higher folding yields of DGK are obtained when PG is included in DDM micelles [33]. More applications are also emerging on the mixed lipid/detergent system used for reversible bR folding. The DMPC/CHAPS mixtures are thought to form disc-shaped micelles, or bicelles, with a small disc of DMPC bilayer surrounded by CHAPS. Such bicelles have now been used to stabilise the apoprotein of the vision receptor rhodopsin, fold membrane receptors and in the crystallisation of the β-adrenergic receptor [34–37]. The size of the bicelle disc seems to be important as does the presence of CHAPS, which may interact specifically with the receptor proteins [34]. The influence of the membrane on protein stability is also being revealed by studies on the energetics of helix–helix interactions (reviewed in [38]). A notable addition here is a comparison of helix–helix interactions in membranes and detergents [39^•^].

## The origin of lipid bilayer effects: a role for monolayer curvature

**Figure 2:**
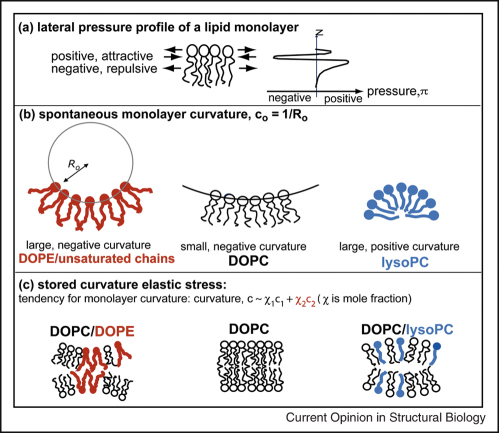
Schematic diagram of relevant lipid bilayer properties. **(a)** The lateral pressure profile [48] of a lipid monolayer rationalises many lipid bilayer properties. Lateral pressures arise parallel to the plane of a monolayer. In the polar to non-polar interface region there is a positive, attractive pressure because of the hydrophobic effect. Negative lateral pressures act in the opposite direction in the headgroup or chain region. An imbalance of pressures within the monolayer causes it to curve away from or towards water as measured by the spontaneous monolayer curvature, *c*_0_**(b)** [49]. This curvature cannot be satisfied in a bilayer, leading to stored curvature elastic stress **(c)**. Phosphatidylcholine (e.g. DOPC) lipids have negligible *c*_0_ and tend to form fluid, lamellar (bilayer) phases. Phosphatidylethanolamine lipids like DOPE have a larger negative *c*_0_ and form non-bilayer phases. Addition of PE to a PC bilayer increases the monolayer curvature (giving a more negative *c*_0_) and the stored curvature elastic stress of the PC/PE bilayer. Single chains (lyso lipids) have large positive curvature away from water and form micelles. Thus, addition of lysoPC to DOPC lowers monolayer curvature (a less negative *c*_0_).

**Figure 3:**
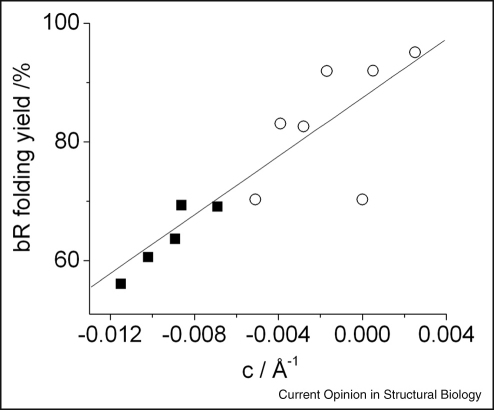
Folding yields for bacteriorhodopsin are a function of lipid bilayer curvature, *c*. bR was refolded into PC vesicles containing ○, lysoPC or ■, PE. Approximate *c* values are obtained by assuming ideal mixing of the lipids and adding the spontaneous curvature (*c*_0_) of the individual lipid components taking into account the lipid mole fraction (*χ*): *c* = *χ*_(*x*)_*c*_0(*x*)_ + (1 − *χ*_(*x*)_)*c*_0(PC)_, where *x* is the added lipid PE or lysoPC. We use *c*_0_ values of the C18:1 lipids for bR, as there are no literature values available for C16:1 chains used in the measurements for bR. We assume that this introduces a systematic error into the bR correlation. Literature spontaneous *c*_0_ values used for DOPC, DOPE and C18:1 lysoPC are: −0.00625 Å^−1^ for DOPC and −0.0188 Å^−1^ for DOPE [49–52]. bR data from [40]. The line is a linear fit to the data, correlation coefficient 0.87.

Elastic properties of the lipid bilayer are vital to the folding and function of membrane proteins [4]. bR folding shows clear dependences on lipid composition that can be interpreted because of changes in the stored bilayer elastic energy [40]. PE lipids decrease folding, whilst single chain lysoPC lipids optimise the folding yield. PE lipids are non-bilayer lipids, owing to a high spontaneous curvature of their monolayers towards water, whilst PC lipids have lower spontaneous curvatures and form lamellar phases (see Figure 2). Incorporating PE into PC bilayers increases the monolayer curvature but as the monolayers are held flat in the bilayer, the stored curvature stress increases. Conversely monolayer curvature can be reduced with a single chain lyso lipid. In order to determine the extent of correlation between folding and spontaneous curvature we estimate the curvature (*c*) of the mixed lipid monolayers used in the earlier bR studies [40]. A clear correlation with these estimated monolayer curvature, *c*, values can be seen in Figure 3 for the folding yield of bR across both PC/PE and PC/lysoPC mixtures. This suggests a strong controlling influence of monolayer curvature on bR folding. Further investigations quantifying the effects of different lipid properties are likely to be illuminating in elucidating the key factors for regulating the folds of proteins within membranes.

## Conclusions and further complexities

The advances described here, exploring stabilising interactions, transition states and folding landscapes, herald a new era in membrane protein folding. The increasing number of high-resolution structures for membrane proteins holds much promise for extending such folding investigations to other more complex proteins. An important area will be the legion of membrane proteins that have more than one domain and operate in heterogenous complexes. An exciting example here is a recent report using electrospray mass spectrometry to investigate an ABC transporter with two membrane-bound and two aqueous subunits [41^••^].

Studies on membrane protein folding ignore the solvent at their peril. Understanding the effects of different lipids, bicelle and bilayer properties continue to provide vital information. Future quantification of these effects promises to elucidate the lipid factors that drive folding as well as provide a link between the wealth of biophysical information on lipid systems and the situation in a biological membrane. There have also been many recent advances in understanding membrane protein biogenesis and the insertion of transmembrane helices by the translocon [42–44], as well as on the role which certain types of lipids have on correct folding and topology of membrane proteins in cells [45]. Moreover, there have been important developments on misfolding and mis-trafficking of membrane proteins in the cell and how this can be partly remedied by chemical agents or pharmacological chaperones [46,47]. The breadth of all this work in biophysics and cell biology offers exciting opportunities for investigations into this infamously elusive and important class of proteins that guard cellular exit and entry points and account for the vast majority of drug targets.

## References and recommended reading

Papers of particular interest, published within the period of review, have been highlighted as:

• of special interest
•• of outstanding interest
